# Factors Associated with the Competencies of Public Health Workers in Township Hospitals: A Cross-Sectional Survey in Chongqing Municipality, China

**DOI:** 10.3390/ijerph121114244

**Published:** 2015-11-09

**Authors:** Zhifei He, Zhaohui Cheng, Hang Fu, Shangfeng Tang, Qian Fu, Haiqing Fang, Yue Xian, Hui Ming, Zhanchun Feng

**Affiliations:** School of Medicine and Health Management, Tongji Medical College, Huazhong University of Science and Technology, No. 13 Hangkong Rd., Wuhan, Hubei 430030, China; E-Mails: houis123@163.com (Z.H.); czhbxt@163.com (Z.C.); tongjifh@163.com (H.F.); shangfenghust@sina.com (S.T.); sofureqian@hotmail.com (Q.F.); fanghaiqing.net@hust.edu.cn (H.F.); bantianxing@126.com (Y.X.); minghuitj@sina.com (H.M.)

**Keywords:** competencies, public health workers, township hospitals, impact factors, Chongqing Municipality

## Abstract

*Purpose*: This study aimed to explore the competencies of public health workers (PHWs) of township hospitals in Chongqing Municipality (China), and determine the related impact factors of the competencies of PHWs; *Methods*: A cross-sectional research was conducted on 314 PHWs from 27 township hospitals in three districts in Chongqing Municipality (China), from June to August 2014. A self-assessment questionnaire was established on the basis of literature reviews and a competency dictionary. The differences in competencies among the three districts were determined by adopting the chi-square test, t-test, analysis of variance (ANOVA) method, and the impact factors of the competencies of PHWs were determined by adopting stepwise regression analysis. *Results*: (1) Results of the demographic characteristics of PHWs in three sample districts of Chongqing Municipality showed that a significant difference in age of PHWs (*p* = 0.021 < 0.05) and the majors of PHWs (*p* = 0.045 < 0.05); (2) In terms of the self-evaluation competency results of PHWs in township hospitals, seven among the 11 aspects were found to have significant differences in the three districts by the *ANOVA* test; (3) By adopting the t-test and *ANOVA* method, results of the relationship between the characteristics of PHWs and their competency scores showed that significant differences were found in the economic level (*p* = 0.000 < 0.05), age (*p* = 0.000 < 0.05), years of working (*p* = 0.000 < 0.05) and title of PHWs (*p* = 0.000 < 0.05); (4) Stepwise regression analysis was used to determine the impact factors of the competencies of PHWs in township hospitals, including the economic level (*p* = 0.000 < 0.001), years of working (*p* = 0.000 < 0.001), title (*p* = 0.001 < 0.005), and public health major (*p* = 0.007 < 0.01). *Conclusions*: The competencies of the township hospital staff in Chongqing Municipality (China), are generally insufficient, therefore, regulating the medical education and training skills of PHWs is crucial to improve the competencies of PHWs in the township hospitals of Chongqing Municipality. The results of this study can be mirrored in other areas of China.

## 1. Introduction

The health system serves a population of 1.3 billion in China, which is the World’s largest population [1]. The primary health care (PHC) system is the main component of China’s health system. Large numbers of studies [2,3] have shown that a good PHC system improves population health and estimates the gaps in health caused by disparities in socioeconomic status. Township hospitals as key providers of PHC play a crucial role in the three-level network of rural public health in China. Currently, township hospitals in China experience human resources shortage and insufficient abilities [4]. The 11th “five-year plan” of health programmers in China includes several strategies that will be beneficial for public health workers at all levels [5]. PHWs are the direct providers of health service, and they determine the quantity, quality, style and cost of public health service [6]. The competencies of PHWs of township hospitals play an irreplaceable role in the primary public health system and the need for competent public health professionals increases day by day [7].

In 1973, McClelland published a paper entitled “Testing for Competence Rather than for Intelligence”, and it indicated that the concept of competencies involves the knowledge, skills, abilities and characteristics of outstanding individuals [8]. McLagan argued that competencies involve individuals who could finish the main job with knowledge, skills and abilities [9]. Boyatzis explained that competency is a kind of ability that could meet the organizational environment needs [10]. Spencer found competency is an individual’s deeper and potential features [11]. Although competency has many definitions, competency generally indicates that an individual’s deeper characteristics can differentiate elitists from ordinary workers, and those characteristics include the motivation, peculiarity, self-image, attitude and value, knowledge, perceived quality, and skills.

Chongqing Municipality is located in Western China. According to a previous study, the ratio per 1000 rural population of township hospitals staff is low in Western China, and in 2013, the ratio of township hospital staff was only 2.15 workers per 1000 rural population [12]. Chongqing Municipality only reached half of the average number of national PHWs in township hospitals [12]. At the same time, the structure of PHWs is inappropriate and personnel utilization was inefficient [13]. Moreover, the loss of staff in township hospitals is serious, with some of the remaining staff lacking work capability [14]. With the development of public health, the personal quality of public health workers is an irreplaceable impact factor of township hospitals in rural China [4,15,16]. The public health workforce is faced with many challenges, such as a diminishing number of workers due to the great number of retiring public health workers [17], job losses associated with budget cuts [18], non-competitive salaries and benefits that cause difficulty in recruiting new workers [18], and limited formal public health training for public health workers among others [19]. Therefore, the need for the competency of PHWs is so urgent that it can influence the performance of township hospitals. According to a study, the concept of competency encompasses the combination of skills, performance, and daily behavior [20]. Competency provides a framework for practice standards designed to assist new practitioners in a specialization to develop necessary skills [21].

Aside from external factors, such as the environment, social culture, and economic level that can influence a hospital’s efficiency, the competencies of PHWs in township hospitals also play a more vital role in influencing a hospital’s efficiency [22]. According to the classification of competency, the health system mainly focuses on leader’s competency, such as the competency of the directors of hospitals [23], the director of the Center for Disease Control (CDC), and the principals of the Community Health Service Centers and township hospitals [24]. However, studies on the core competencies and professional competencies of PHWs in township hospitals in China are limited [25]. The competencies of PHWs in township hospitals in rural China are so vital that they can decide the development level of one township hospital [26]. This study focuses on the competencies of PHWs of township hospitals in Chongqing Municipality (China), and determines the relative impact factors of the competencies of PHWs. The competencies not only improve the professional abilities and skills of PHWs in sample areas but also serve as reference for enhancing the working abilities of PHWs of other township hospitals in rural China.

## 2. Methods

### 2.1. Sample Method

This cross-sectional survey was conducted from June to August 2014 in Chongqing Municipality (China). A multistage stratified purposive sampling method was adopted (Table 1). 

**Table 1:** Sampling strategy and achieved sample size by area.

Province	Districts	Income Level	Participating Hospitals	Sample Size PHWs (%)	Total PHWs Size (%)
Chongqing Municipality	Jiulongpo	Developed	9 township hospitals	100(31.85)	118(34.40)
	Qianjiang	Middle economic level	9 township hospitals	107(34.08)	110(32.07)
	Hechuan	Underdeveloped	9 township hospitals	107(34.08)	115(33.53)

The regions surveyed were divided into three types of districts based on economic conditions [27], including developed district (Jiulongpo), district of middle economic level (Qianjiang), and economically underdeveloped district (Hechuan). Nine township hospitals were selected in each sample district, and the total number of township hospitals was 27. A questionnaire survey was conducted on PHWs in the 27 township hospitals (343 PHWs). The valid number of sample PHWs was 314.

### 2.2. Survey Content

A “Competencies of PHWs of Township Hospitals” questionnaire was designed. The questionnaire included two parts: the demographic characteristics of the PHWs in township hospitals (*i.e*., name, age, gender, educational background, major, and title) and the competencies of PHWs. According to some related studies and a competency dictionary [28,29,30,31], we selected 11 aspects of competencies from 20 aspects. We designed two relevant questions for each aspect. Overall, 22 questions of 11 aspects were obtained. Based on the Likert scale, we designed four alternative options for each question, with each option representing a score (definitely low, 1 score; low, 2 score; possible high, 3 score; high, 4 score). A higher score represented a higher level. The highest score was 88 points (full score), whereas the lowest score was 22 points. We tested the second part of the questionnaire, and the Cronbach’s alpha coefficient was 0.808. The reliability of the questionnaire was acceptable because the Cronbach’s alpha coefficient is higher than 0.7 [32,33], and the questionnaire was recognized by health professors.

### 2.3. Survey Methodology

Trained and qualified investigators were separately sent to each township hospital. The personnel in charge of the PHS gathered the health workers in the township hospitals, and then the investigators described the purpose of the survey to the hospital staff. Those who agreed to take part in the survey were supplied with the (anonymous) self-administered questionnaires. Completed questionnaires were collected and checked at the scene.

### 2.4. Statistical Analysis

Data entry was performed using Excel and Epidata. A double-entry method was adopted to ensure the accuracy of the data. SPSS 19.0 statistical software (Version 19.0; SPSS Inc., Chicago, IL, USA) was used for statistical analysis. The factors of PHWs in township hospitals were identified by the descriptive statistics method. The competencies of PHWs in three districts were the compared using the chi-square test, *ANOVA* and *t*-test. The associated impact factors of the competencies of PHWs were identified by adopting the stepwise regression analysis method. The stepwise regression analysis model belongs to multiple linear regression model, which determined the dependent relationship among variables. According to related research, we consider the score of self-evaluation competencies as the dependent variable and the factors of economic level, years of working, title and major as the independent variables [15].

### 2.5. Results

#### Characteristics of the Sample

Characteristics of the study sample are shown in Table 2. Among the 343 survey questionnaires of PHWs in the 27 township hospitals, 314 (91.55%) were completed and 29 were incomplete. Among the 314 respondents, 161 (51.3%) were male and 153 (48.7%) were female PHWs. In terms of location, 100 PHWs were in Jiulongpo District, 107 in Qianjiang District and 107 in Hechuan District. No significant difference was found among the three districts (*p* = 0.883 > 0.05) in terms of gender. A significant difference was found among the four age groups (*p* = 0.021 < 0.05). Among the PHWs, 110 (35.0%) were aged 31 to 40 years, and 113 (36.0%) were aged 41 to 50 years. These two age groups accounted for the largest proportion among the four age groups. Specifically, the 31–40 age group accounted for the largest proportion among the four age groups (45.0%) in Jiulongpo, and the 41–50 age group accounted for the largest proportion in Qianjiang and Hechuan, at 35.5% and 42.1%, respectively. We divided years of working into four groups, and no significant difference was found among them (*p* = 0.195 > 0.05). Two groups had the largest ratio: the group of 107 (34.1%) PHWs who worked for less than 10 years, and the group of 114 (36.3%) PHWs who worked from 11 to 20 years. No significant difference was found among the three educational background groups (*p* = 0.369 > 0.05). Among the PHWs, 107 (34.1%) had a junior high school educational degree, 119 (37.9%) had a senior high school educational degree, and 88 (28.0%) had a college educational degree. The PHWs had five groups of majors, with 27.1% of them specializing in clinical work, 25.8% in nursing and 24.2% in public health (full-time public health workers in township hospitals). A significant difference was found among the five major groups (*p* = 0.045 < 0.05). In particular, the number of public health majors was the largest (33.0%) in Jiulongpo District, and the number of clinical majors was the largest in Qianjiang and Hechuan, at 27.1% and 29.9%, respectively. The title of the PHWs focused on the junior and middle levels, for a total rate of 75.2%. Among the PHWs, 13 (4.1%) had a senior qualification title, and 65 (20.7%) did not have any title. No significant difference was found among them (*p* = 0.142 > 0.05).

### 2.6. Self-Evaluation Competencies of PHWs in Township Hospitals

In terms of the self-evaluation competencies of PHWs in township hospitals, we adopted the *ANOVA* method to analyze the scores of PHWs in the three districts (Table 3). The 11 aspects were achievement orientation (ACH), performance orientation (PMO), detailed test objectives (DTO), action (ACT), learning (LRN), flexible and adaptive (FAA), teamwork (TMW), public relations ability (PLR), focusing on the service objectives (CMF), communication (CMC), and responsibility (RSP). Each aspect has two related titles, and each question has a score. In this study, we calculated the mean sores and compared them among the three different districts using *ANOVA*. Among the 11 aspects (22 questions), seven aspects had mean scores with significant differences: PMO (*p =* 0.026 < 0.01), ACT (*p =* 0.002 < 0.01), LRN (*p =* 0.013 < 0.01), FAA (*p =* 0.002 < 0.01), PLR (*p =* 0.005 < 0.01), CMF (*p =* 0.000 < 0.001), and CMC (*p =* 0.002 < 0.01). In the aspect of PMO, the mean score of competencies of PHWs in Jiulongpo was higher than that of PHWs in Hechuan (①>③). The mean score of ACT competencies of PHWs in Jiulongpo was higher than that of PHWs in Qianjiang. The mean score of ACT competencies of PHWs in Hechuan was higher than that of PHWs in Qianjiang, but no significant difference was found between Qianjiang and Hechuan (①>②, ③>②). The mean score of LRN competencies was higher in Jiulongpo than those in Qianjiang and Hechuan. No significant difference was found between Qianjiang and Hechuan (①>②, ①>③). As shown in Table 3, significant differences were found in the mean scores of FAA competencies, PLR competencies, and CMC competencies. (①>②, ①>③, *p* < 0.01). The mean score of CMF competencies was higher in Jiulongpo than that in Qianjiang and Hechuan (①>②, ①>③, *p* < 0.001). Overall, significant differences were found in the PHWs’ competency scores among the three districts (*p* = 0.000<0.005; ①>②, ①>③).

**Table 2:** Demographic characteristics of the PHWs in township hospitals.

Characteristics	Total		Jiulongpo		Qianjiang		Hechuan		*p*
	*N* = 314	%	*N* = 100	%	*N* = 107	%	*N* = 107	%	
Gender									
Male	161	51.3%	53	53.0%	53	49.5%	55	51.4%	0.883
Female	153	48.7%	47	47.0%	54	50.5%	52	48.6%	
Age									
20–30	73	23.2%	22	22.0%	25	23.4%	26	24.3%	0.021 *
31–40	110	35.0%	45	45.0%	32	29.9%	33	30.8%	
41–50	113	36.0%	30	30.0%	38	35.5%	45	42.1%	
51–60	18	5.7%	3	3.0%	12	11.2%	3	2.8%	
Years of working									
1–10	107	34.1%	37	37.0%	41	38.3%	29	27.1%	0.195
11–20	114	36.3%	39	39.0%	30	28.0%	45	42.1%	
21–30	82	26.1%	22	22.0%	30	28.0%	30	28.0%	
31–40	11	3.5%	2	2.0%	6	5.6%	3	2.8%	
Educational background									
Junior high school	107	34.1%	26	26.0%	41	38.3%	40	37.4%	
Senior high school	119	37.9%	37	37.0%	40	37.4%	42	39.3%	0.369
College/University	88	28.0%	37	37.0%	26	24.3%	25	23.4%	
Major									
Clinic	85	27.1%	24	24.0%	29	27.1%	32	29.9%	0.045 *
Nursing	81	25.8%	22	22.0%	28	26.2%	31	29.0%	
Public health	76	24.2%	33	33.0%	24	22.4%	19	17.8%	
Maternal	43	13.7%	11	11.0%	18	16.8%	14	13.1%	
Others	29	9.2%	10	10.0%	8	7.5%	11	10.3%	
Title									
None	65	20.7%	12	12.0%	21	19.6%	32	29.9%	0.142
Junior	134	42.7%	42	42.0%	47	43.9%	45	42.1%	
Middle	102	32.5%	41	41.0%	34	31.8%	27	25.2%	
Senior	13	4.1%	5	5.0%	5	4.7%	3	2.8%	

The *p* value was calculated by chi-square test, * p < 0.05.

**Table 3:** The scores of self-evaluation competencies of PHWs in township hospitals.

Aspects	Questions	Jiulongpo ①(High)		Qianjiang ②(Middle)		Hechuan ③(Low)		*p*	LSD
		Mean	SD	Mean	SD	Mean	SD		
ACH	Inner motivation	3.60	0.49	3.64	0.48	3.67	0.47	0.549	
	High standard	3.72	0.45	3.71	0.46	3.68	0.47	0.826	
	*Mean*	7.32	0.72	7.36	0.66	7.36	0.77	0.922	
PMO	Performance be tracked	3.72	0.45	3.60	0.49	3.57	0.50	0.062	①>③ **
	performance be enhanced	3.18	0.39	3.18	0.47	3.07	0.54	0.131	
	*Mean*	6.90	0.61	6.78	0.73	6.64	0.74	0.026 **	
DTO	Details	3.50	0.50	3.22	0.42	3.38	0.49	0.000	
	Precise working	3.19	0.39	3.30	0.46	3.23	0.43	0.183	
	*Mean*	6.69	0.60	6.52	0.63	6.62	0.64	0.158	
ACT	Initiative	3.24	0.43	3.17	0.38	3.19	0.39	0.410	①>② ***,③>② **
	Impetus	3.81	0.39	3.58	0.50	3.76	0.43	0.001	
	*Mean*	7.05	0.61	6.75	0.60	6.94	0.64	0.002 **	
LRN	Summary of experience	3.84	0.37	3.79	0.41	3.76	0.43	0.336	①>② **,①>③ **
	Gap analysis	3.80	0.40	3.60	0.49	3.67	0.47	0.007	
	*Mean*	7.64	0.58	7.39	0.67	7.43	0.67	0.013 **	
FAA	Decision making	3.47	0.50	3.43	0.50	3.50	0.50	0.550	①>② **,①>③ **
	Frustration tolerance	3.86	0.38	3.55	0.57	3.56	0.54	0.000	
	*Mean*	7.33	0.59	6.98	0.81	7.07	0.80	0.002 **	
TMW	Role adjustment	3.32	0.47	3.28	0.45	3.20	0.42	0.126	
	Technical support	3.37	0.49	3.27	0.45	3.35	0.48	0.284	
	*Mean*	6.69	0.72	6.55	0.62	6.54	0.70	0.222	
PLR	Interpersonal influence	3.44	0.50	3.32	0.47	3.34	0.47	0.148	①>② **,①>③ **
	Conflict management	3.49	0.50	3.31	0.46	3.38	0.49	0.027	
	*Mean*	6.93	0.66	6.63	0.68	6.72	0.70	0.005 **	
CMF	Concern service objects	3.72	0.45	3.56	0.50	3.55	0.50	0.021	①>② ***,①>③ ***
	Develop service objects	3.88	0.33	3.64	0.48	3.68	0.47	0.000	
	*Mean*	7.60	0.55	7.21	0.74	7.23	0.67	0.000 ***	
CMC	Positive interaction	3.80	0.40	3.78	0.42	3.80	0.40	0.862	①>② **,①>③ **
	Mechanism assurance	3.31	0.46	3.08	0.28	3.11	0.32	0.000	
	*Mean*	7.11	0.62	6.86	0.46	6.92	0.48	0.002 **	
RSP	Responsibility cognition	3.28	0.45	3.18	0.38	3.23	0.43	0.215	
	Consciousness	3.73	0.45	3.72	0.45	3.73	0.45	0.983	
	*Mean*	7.01	0.72	6.90	0.66	6.96	0.71	0.504	
Total		78.27	2.67	75.92	3.35	76.42	2.50	0.000 ***	①>② ***,①>③ ***

The mean scores were test with ANOVA method. * *p* < 0.05, ** *p* < 0.01, *** *p* < 0.001.

### 2.7. Relationship between the Factors of PHWs and Self-Evaluation Competencies

In terms of the relationship between the factors (independent variables) of PHWs and the self-evaluation competencies of PHWs (Table 4), the results indicated that gender and educational background had no significant differences. A positive relationship was found between age and self-evaluation competencies of PHWs (F = 13.11, *p* = 0.000 < 0.001). The 51–60 age group obtained the highest score. A significant difference was found between years of working and the self-evaluation competencies of PHWs (F = 15.51, *p* = 0.000 < 0.001). The years of working of the 31–40 age group obtained the highest score. For the majors of PHWs, professional public health workers had the highest score (F = 3.91, *p* = 0.004 < 0.001).in terms of the title of PHWs, public health workers who had an advanced title obtained the highest score among the four groups (F = 11.64, *p* = 0.000 < 0.001).

**Table 4:** Relationship between factors of PHWs and self-evaluated competencies.

Items	*n*	*Mean*	*SD*	*t/F Value*	*p*	*LSD*
Sex						
Male	161	76.88	2.91	*t* = 0.265	0.415	
Female	153	76.79	3.16			
Economic level						
①Hechuan	107	76.42	2.499	F = 19.13 ***	0.000	③>①,③>②
②Qianjiang	107	75.92	3.354			
③Jiulongpo	100	78.27	2.666			
Age						
①20–30	73	75.22	3.42	F = 13.711 ***	0.000	④>②,③,①
②31–40	110	77.19	2.85			
③41–50	113	77.12	2.45			
④51–60	18	79.44	2.87			
Years of working						
①1–10	107	75.51	3.39	F = 15.507 ***	0.000	④>②,③,①
②11–20	114	77.46	2.71			
③21–30	82	77.23	2.07			
④31–40	11	80.27	3.17			
Educational background						
①Junior high school	107	77.37	2.36	F = 2.583	0.077	/
②Senior high school	119	76.53	2.99			
③College/ University	88	76.60	3.68			
Major						
①Clinic	85	76.55	2.68	F = 3.907 ***	0.004	③>④,①,⑤,②
②Nursing	81	76.17	2.79			
③Public health	76	77.93	3.17			
④Maternal	43	76.95	2.92			
⑤Others	29	76.48	3.78			
Title						
①None	65	75.49	2.94	F = 11.641 ***	0.000	④>③,②,①
②Junior	134	76.68	3.04			
③Senior	102	77.51	2.68			
④Advanced	13	79.92	2.50			

The significant differences were tested with t-test and ANOVA analysis, *** *p* < 0.001.

### 2.8. Impact Factors of Self-Evaluation Competencies

Stepwise regression was used to further analyze the factors affecting the competencies of PHWs. Results are shown in Table 5. Related literature showed that the factors associated with the general competencies by the multivariable model—age group, education, years of working and title—are the independent variables [34,35,36]. In this study, the economic level of the three districts (*p* = 0.000 < 0.001), years of working of PHWs (*p* = 0.000 < 0.001), title of PHWs (*p* = 0.001 < 0.005) had significant differences with the self-evaluation competencies of PHWs. In terms of majors, the public health major indicated a significant difference (*p* = 0.007 < 0.01).

**Table 5:** Stepwise regression analysis of factors related to self-evaluation competencies (*N* = 314).

Variables	B	Std. Error	β	*t* value	*p*	95.0% Confidence Interval for B		R^2^	F
						Lower Bound	Upper Bound		
Constant	71.725	0.608		118.047 ***	0.000			0.216 *	21.221 ***
Economic level	0.786	0.195	0.211	4.031 ***	0.000	0.403	1.170		
Years of working	0.925	0.186	0.263	4.961 ***	0.000	0.558	1.291		
Title	0.672	0.201	0.180	3.344 ***	0.001	0.276	1.067		
Major									
*Nursing*			−0.092	−1.710	0.088				
*public health*	0.983	0.361	0.139	2.722 **	0.007	0.272	1.694		
*Maternal*			0.068	1.288	0.199				
*Others*			0.033	0.635	0.526				

* *p* < 0.05, ** *p* < 0.01, *** *p* < 0.001.

## 3. Discussion

The results in Table 2 show that a significant difference exists between age and majors. The results indicated that in terms of the different economic levels, Jiulongpo has the highest economic level among the three districts [37], and this condition may attract more young PHWs because of the higher salary. Moreover, most PHWs work in public health [6]. Apparently, economic level is a vital impact factor of the self-evaluation competency scores of PHWs. The economic level of Jiulongpo is higher than those of Qianjiang and Hechuan. Therefore, PHWs in Jiulongpo have a higher salary and a more positive working attitude than those in other districts, and thus they obtained the highest scores in competencies. Economic level can be a standard to measure public health development. It definitely made the PHWs obtain higher scores [38]. Therefore, improving the economic development of an area can improve the competencies of PHWs.

Competency is defined as “the ability to do something successfully;” that is, the ability to complete a job efficiently [22]. Table 3 shows the significant differences in the competencies of PMO, ACT, LRN, FAA, PLR, CMF, and CMC among the three sample districts. In terms of PMO, Jiulongpo is better than Hechuan at the economic level, and the scores of PHWs’ competencies in Jiulongpo are higher than those of PHWs in Hechuan. According to the literature, PMO involves “performance to be tracked” and “performance to be enhanced.” To some extent, PMO decides the public health working effect of PHWs, especially in township hospitals the working performance of which is associated with their economic level [11,39]. Reasonable and effective motivation mechanisms can improve health care performance by influencing the PHWs’ behaviors [4]. Similar to the results, the higher the economic level is, the higher the competency scores of the PHWs.

The competencies of ACT of PHWs in Jiulongpo and Hechuan are higher than those of PHWs in Qianjiang. As McClelland argued, “competencies rather than intelligence” [8]. ACT includes initiative and impetus, that is, communicating with leaders and co-workers positively, and not delaying tasks. In this study, Jiulongpo township hospitals have the most excellent PHWs, who also have the highest scores in ACT competency. Although Hechuan township hospitals have poorly performing PHWs, these PHWs may have stronger competencies rather than intelligence. Thus, they could also obtain high scores in ACT competency.

The scores of LRN competency of PHWs in Jiulongpo were higher than those of PHWs in Qianjiang and Hechuan. No significant differences were found between Qianjiang and Hechuan. The reasons for this result are that Jiulongpo township hospitals have more excellent PHWs and that training and learning can enhance the skills of PHWs by learning from failed experiences [40,41]. A number of studies have shown similar results. According to Leung, competency-based training is the progression referenced to the demonstrated ability to perform certain tasks [42]. In our study, the scores of the LRN competency of PHWs in Jiulongpo were higher than those of the LRN competency of PHWs in the other two sample districts. Therefore, the PHWs in Jiulongpo district may obtain more effective training and experience.

The competencies of FAA, PLR, CMF, and CMC have significant differences among the three districts (①>②, ①>③; *p* < 0.01). As shown in Table 3, the scores of competencies of FAA, PLR, CMF and CMC of PHWs in Jiulongpo were higher than those of the PHWs in Qianjiang and Hechuan. According to Al-Njjar and Alsouyf, FAA included “decision making” and “frustration tolerance”, that is, adjusting the strategies for different cultures and people [43]. The capability in public health of the PHWs or the attitude of PHWs to residents’ unreasonable response reflects public health in rural China [44]. Meanwhile, the working ability of PHWs indicates their knowledge and professional training in township hospitals. According to Sportsman and Fleig-Palmer, PLR involved “interpersonal influence” and “conflict management” [45,46], which verify how the PHWs in Jiulongpo District always take effective measures to solve problems. According to Wing, CMC means responsibility and consciousness. That is, communicating and establishing communication mechanism and CMF include positives communication, which predicts the demands and creates values for service objects [47]. In this study, the results prove that the more the PHWs are responsible and communicate with each other, the higher the scores they will obtain [48].

Rapidly developing the primary health care system and improving the level of primary health care quality are the vital tasks of the new health reform plans in China. Township hospitals are considered to be the bridge of primary health care and play a key role. Therefore, improving the competencies of PHWs in township hospitals is a priority. In this study, we selected 11 aspects based on a competency dictionary and related literature, with each aspect playing a significant role. ACH and PMO are the guides among the 11 aspects that provide their targets. Through DTO, ACT, LRN, FAA, TMW, PLR, CMF and CMC we can gain RSP and even acquire great achievement and better performance in enhancing the competencies of PHWs and improving public health performance.

The analysis in Table 4 indicate the relationship between the competencies and the factors using *t*-test and *ANOVA*. Further study on the impact factors using stepwise regression analysis is presented in Table 5. The results show five relevant factors of the PHWs’ self-evaluation competency scores: economic level, age, years of working (yeas as a PHW), major, and title [49]. As previously mentioned, a higher economic level causes higher competency scores. As shown in Table 4 and Table 5, the self-evaluation competency score of the PHWs in Jiulongpo is higher that of the PHWs in Qianjiang and Hechuan. A consistent relationship exists among “age”, “years of working”, and “title”. This result indicates that the longer the years of working are, the older the PHWs and the higher their titles. Years of working is an important impact factor of competency [50]. The more years of work experience PHWs have, the higher their score in self-evaluation competencies [15]. Years of working are correlated with one’s working experience. Therefore, the longer the years of working PHWs have, the more working experience they have. Working experience is a kind of education that can be obtained from outside the workplace or from the work itself. Training is usually a way of increasing working experience and improving competencies [51]. A number of studies have shown similar results. According to Liou, a significant difference exists between PHW’s years in public health and clinical competencies, that is, the higher the number of working years of PHWs is, the higher their average score in public health than in clinical competencies [52]. A richer work experience obtains a higher self-evaluation competency score. The different titles of PHWs have different self-evaluation competency scores. Clearly, PHWs who have a higher title get a higher competency score. According to Wu, public health institutions cannot supply high-quality service without a higher education level of their PHWs [5]. In other words, most PHWs with advanced titles have a higher educational level. In addition, a higher educational level adds to the rich working experience of PHWs so that they will become better at their job. PHWs who work in the public health service have a higher competency score than others. This findings indicates that PHWs who work in public health service possessed more professional knowledge than those who do not.

In terms of the majors of PHWs, the professional public health workers obtained the highest score among the five groups, and they play a vital role in the public health service of township hospitals [53]. A previous study showed that public health education has become part of medical education and that is has its own system and scale, and has played an important role in the public health field [48].

## 4. Conclusions

In this study, we were able to identify the competencies of PHWs in township hospitals in Chongqing Municipality in Western China that needed to be improved. The results of this study indicate some factors influencing the competencies of PHWs in township hospitals, such as economic level, age, years of working, title and major.

In light of the above, some proposals may be put forth. First, from the perspective of national policy, the Chinese government should establish related policies for township hospitals and primary health care. Second, the structure of PHWs should be optimized to provide more outstanding PHWs for the public health system in China. Last but not least, the knowledge, skills, and attitudes of PHWs should be improved through variety of training forms to improve the competencies of PHWs and the residents’ satisfaction at the same time.

## 5. Limitation

The results came from 314 PHWs of 27 township hospitals from three districts in Chongqing Municipality (China). As the sample size is not large, it perhaps cannot represent the condition of all the township hospitals’ PHWs in rural Western China. Nevertheless, the average number of PHWs of each township hospital is small according to the Chinese national situation, and Chongqing Municipality is not exempted, as the average number of PHWs (total number of full-time and part-time PHWs) in each township hospital is no more than 13. On the one hand, the sample size can represent the entire condition of PHWs of township hospitals in Chongqing Municipality. On the other hand, the results may reflect the condition of other PHWs of township hospitals in rural Western China to some extent. To ensure that our research result more precisely reflects the condition of PHWs of township hospitals in rural China, we will conduct a further study on township hospitals’ PHWs in other regions such as the Middle regions and Eastern regions of China in the future. We designed the questionnaire by ourselves. Although it was tested by related professions, it still has problems. For instance, some questions need to be simplified. Moreover, the subjective answers of some PHWs to some questions led to partial information, which influenced the quality of the questionnaires more or less.

## Abbreviations

PHWs: Public Health Workers
PHS: Public Health Service
ACH: Achievement orientation
PMO: Performance orientation
DTO: Detailed test objectives
ACT: Action
LRN: Learning
FAA: Flexible and adaptive
TMW: Teamwork
PLR: Public relation ability
CMF: Focusing on the service objectives
CMC: Communication
RSP: Responsibility
